# Impacts on study design when implementing digital measures in Parkinson's disease-modifying therapy trials

**DOI:** 10.3389/fdgth.2024.1430994

**Published:** 2024-10-09

**Authors:** Jennie S. Lavine, Anthony D. Scotina, Seth Haney, Jessie P. Bakker, Elena S. Izmailova, Larsson Omberg

**Affiliations:** Research & Development, Koneksa Health, New York, NY, United States

**Keywords:** Parkinson's disease, digital health technology, measurement reliability, clinical trials, statistical power, disease progression, longitudinal data, simulation study

## Abstract

**Introduction:**

Parkinson's Disease affects over 8.5 million people and there are currently no medications approved to treat underlying disease. Clinical trials for disease modifying therapies (DMT) are hampered by a lack of sufficiently sensitive measures to detect treatment effect. Reliable digital assessments of motor function allow for frequent at-home measurements that may be able to sensitively detect disease progression.

**Methods:**

Here, we estimate the test-retest reliability of a suite of at-home motor measures derived from raw triaxial accelerometry data collected from 44 participants (21 with confirmed PD) and use the estimates to simulate digital measures in DMT trials. We consider three schedules of assessments and fit linear mixed models to the simulated data to determine whether a treatment effect can be detected.

**Results:**

We find at-home measures vary in reliability; many have ICCs as high as or higher than MDS-UPDRS part III total score. Compared with quarterly in-clinic assessments, frequent at-home measures reduce the sample size needed to detect a 30% reduction in disease progression from over 300 per study arm to 150 or less than 100 for bursts and evenly spaced at-home assessments, respectively. The results regarding superiority of at-home assessments for detecting change over time are robust to relaxing assumptions regarding the responsiveness to disease progression and variability in progression rates.

**Discussion:**

Overall, at-home measures have a favorable reliability profile for sensitive detection of treatment effects in DMT trials. Future work is needed to better understand the causes of variability in PD progression and identify the most appropriate statistical methods for effect detection.

## 1 Introduction

Parkinson's Disease (PD) is a slow-progressing neurodegenerative disease that affects over 8.5 million people worldwide and is currently the fastest growing neurodegenerative disease in the world (1). Hallmarks of PD include slowness of movement and rigidity, and the impacts are felt in many aspects of everyday motor function including gait, eating, speech, and dressing. Currently available PD medications address symptoms but do not treat the underlying disease. Recent advances in drug development show promise for disease modifying therapies (DMTs) but evaluation of these treatments is hampered by outcome measures such as the Movement Disorder Society-Unified Parkinson's Disease Rating Scale (MDS-UPDRS), which requires large sample sizes and/or long term follow-up to detect modest treatment effects, especially given that existing symptomatic treatment can mask underlying progression (2). Digital at-home measures, which allow for more frequent assessment, are a promising option for detecting treatment effects in shorter timeframes and/or with a smaller number of participants.

Digital measures are currently recommended as exploratory endpoints in randomized controlled trials (RCTs) (3). For use as primary and secondary endpoints, and regardless of whether the measure is considered a biomarker or a clinical outcome assessment, a better understanding of their reliability and responsiveness to disease progression is necessary to determine their optimal context of use and assessment schedule. Clinimetric properties of digital tools have been assessed in a wide range of studies to determine how they can be useful in PD (see Supplementary Table 1 and references within). Multiple studies of digital measures derived from at-home app-based assessments, such as finger tapping and timed walk tests, demonstrate associations with aligned in-clinic assessments and high test-retest reliability [(4–7), Supplementary Table 1]. The reliability of many of these measures is as good as or better than test-retest reliability for MDS-UPDRS part III scores (8).

In current clinical trials for novel DMTs for PD, the MDS-UPDRS or one of its subparts is the gold standard outcome measure (3). Composed of four parts, each of which consists of multiple items scored ordinally from 0 to 4 (where 0 is no symptoms and 4 is severe symptoms), the items comprise patient-reported outcomes and clinician assessments (9). Parts II and III relate to motor function, measuring patient perception and clinician ratings of motor impacts respectively. These parts have excellent test-retest reliability as measured by intraclass correlation coefficients (ICCs) across spans of 1−2 weeks [ICCs for part II: 0.96, part III: 0.93 (8)]; however, it remains challenging to detect changes in early disease burden, especially in the face of symptomatic treatments (2). One explanation for this apparent conundrum is that there are three fundamentally different sources of variability in measurements of PD motor function: measurement error, short-term clinical fluctuations, and long-term variability in underlying disease progression.

On the timescale of days to a few weeks, there is no expectation of change in underlying disease severity, yet measures vary from one time point to the next due to measurement error and day-to-day fluctuations in symptoms. Measurement error may be present in clinician ratings due to, for example, interrater reliability (10, 11) and in at-home digital assessments due to, for example, variability in the setting in which patients use the digital devices assessments (12). Also on a short time scale, clinical variability results from day-to-day and diurnal symptom fluctuations including those induced by levodopa and other symptomatic treatment medications (13). These types of variability can be quantified with the ICC, standard error of measurement (σ_m_), and minimum detectable change (MDC) in cross-sectional studies and have been established for both in-clinic and at-home assessments.

In contrast, long-term variability in underlying disease progression arises from PD being a heterogeneous disease. When averaged over individuals, the progression of PD motor manifestations as measured by MDS-UPDRS or digital assessments can be approximated as linear over the span of a year or two (2, 14). However, PD's motor manifestations do not change at a constant rate across months within (2, 15) or between (16, 17) individuals. The causes of inter- and intra-individual variability in disease progression are not well known and may include differences in underlying disease etiology, seasonality, stress, climate, and changes in living situation (15, 16). Variability in progression rate is harder to estimate because it is only apparent at long timescales; however, it is detectable in longitudinal MDS-UPDRS data such as those collected in the PPMI study (18) and has been disentangled from measurement error by Evers et al. (15).

Digital assessments can help overcome the challenges posed to clinical trials by all three of these types of variability by allowing for more frequent measures. Including repeat measures reduces the standard error of endpoint estimates such as the rate of change from baseline. In contrast with clinician-observed outcome assessments, which are typically captured infrequently due to the burden and cost of clinic visits, the schedule of assessments for digital measures can be driven by study designs that yield the highest power for detecting the treatment effect.

Multiple outcome measures have been considered from assessments completed using digital tools. These include individual measures, such as number of taps or gait speed derived from a mobile app-based assessment, and summary statistics of a burst of the same assessment, such as the median of 6 tapping assessments completed over the course of seven days. There is a trade-off between these two outcome measures: individual measures can be completed more frequently, but median values of bursts have higher test-retest reliability (7).

While digital measures have been used in clinical trials as exploratory endpoints, it remains unclear under what conditions they will outperform in-clinic assessments and how best to distribute assessments across the length of the trial to detect the treatment effect. We undertook analyses to address these gaps with the following objectives: (1) Estimate measurement error in a variety of at-home digital assessments spanning gait, tapping, and tremor, which are part of a neuroscience toolkit developed by Koneksa Health for use in clinical trials. The measures, derived from raw triaxial accelerometry sensor data (19), were applied to data collected in the Objective PD sub-study of the mPower study (20). (2) Simulate various DMT study designs that implement individual measures and bursts using at-home digital assessments vs. in-clinic MDS-UPDRS. We use the Gaussian state space framework developed by Evers et al. (15), which explicitly models measurement error and variability in disease progression rates. (3) Assess the power to detect a treatment effect in the various scenarios by fitting linear mixed effects models to the simulated measures.

## 2 Methods

### 2.1 Data

The data used in this study to estimate reliability of digital assessments derive from the ObjectivePD sub-study (20), which recruited 44 participants (21 with confirmed PD diagnosis, 23 healthy controls). Participants were followed for 6 months and seen in clinic three times at 0, 3 and 6 months. During the entire 6 months, they were also asked to complete daily digital health measures administered through the mPower mobile application (20). These assessments consisted of (1) speeded finger tapping alternating between the index and middle finger, (2) a 30-s walk test with the phone in the pocket, and (3) three tremor assessments including resting, postural and hand-to-nose tremor. Each participant in the ObjectivePD sub-study performed on average 182 tapping sessions, 147 gait assessments, and 134 tremor sessions throughout the 6 months study timeframe. Additional details of the measures are available in prior publications (20, 21) and Supplementary Table 2.

### 2.2 Reliability measure estimation

We estimated measurement error and test-retest reliability of at-home digital measures using a linear random intercept model. We assessed the test-retest reliability of measures derived from individual at-home assessments and measures that summarize multiple at-home assessments completed within a 7-day period with their median. Specifically, at-home measurements assessed longitudinally per participant were grouped by fortnight, and a linear model was fit per digital measure with random intercepts for participant and participant-by-fortnight interaction. In contrast with conventional methods for calculating test-retest reliability that rely on two parallel assessments (e.g., assessments taken on the same participant over a short period of time, or assessments collected from two raters at the same point in time), assessment of test-retest reliability with a longitudinal model uses all measurements collected during the study and are robust to missing data (22). Furthermore, test-retest reliability in this context can be interpreted as the consistency between measurements collected during any 2-week period. Implicit in this calculation is the assumption that underlying disease progression between observations within a fortnight will be minor (8). This analysis was performed separately for measures that summarized bursts and measures that represented individual assessments. Model residuals were plotted to assess whether the model was an appropriate choice.

For each fitted model, we extracted the measurement error associated with a particular measure as the residual variance, σ^2^*_m_*. Test-retest reliability, assessed with the intraclass correlation coefficient, is extracted from the fitted model; it is the proportion of the overall variability in a digital measure explained by the participant effect and the participant-by-fortnight interaction effect.

We calculated the minimum detectable change (MDC) associated with each digital measure following Weir (23) as:

$$\mathrm{MDC}\;=\;1.96\times \sqrt{2}\times \sigma_{m}$$

### 2.3 Model for simulating digital and in-clinic data

**Figure 1:**
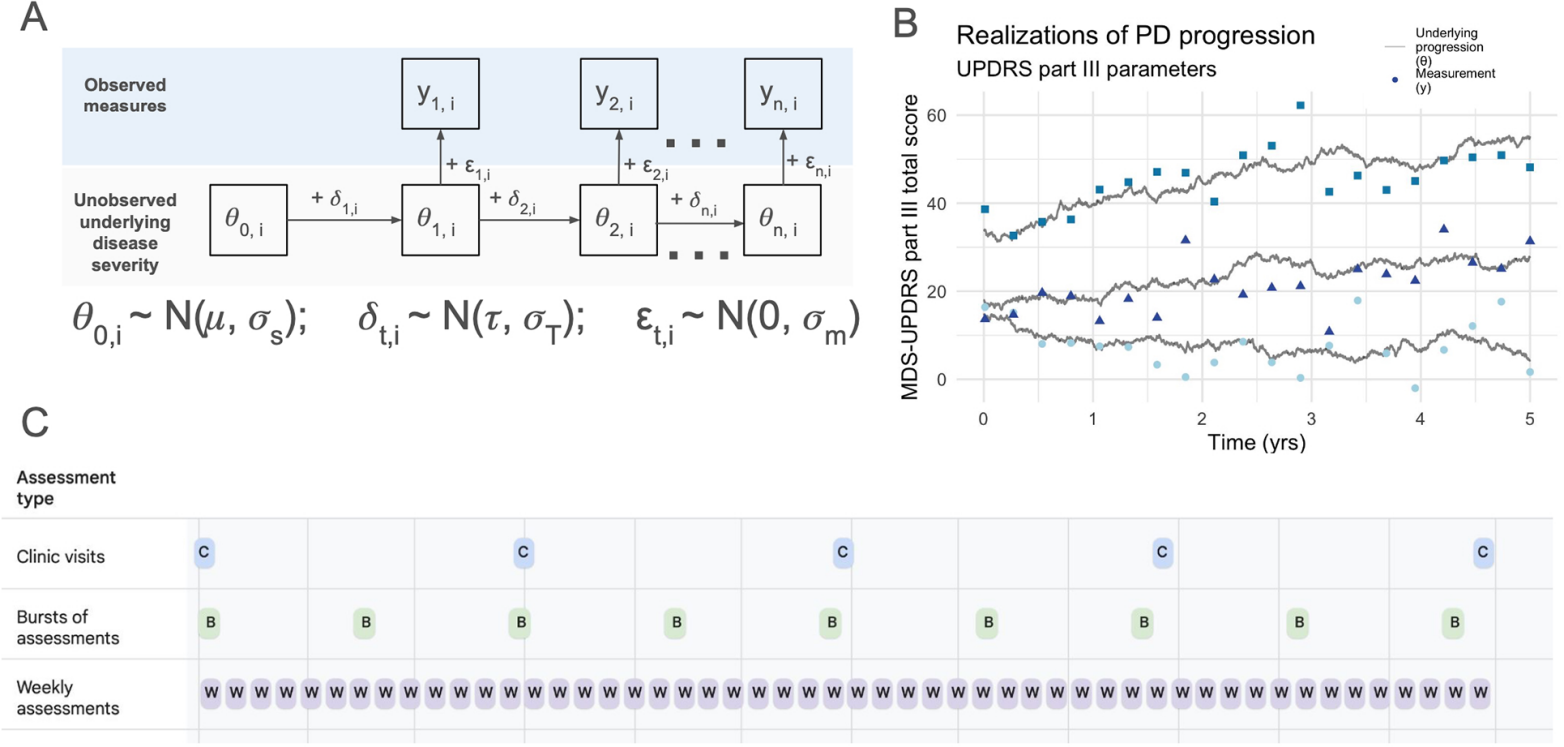
Conceptual model and simulation framework. **(A)** Visual representation of the Gaussian state space model used for simulations. **(B)** Three stochastic realizations of the model, using in-clinic MDS-UPDRS part III parameters (see Table 1). Underlying disease progression is represented by gray lines in **(B)** and $\theta_{t,i}$ in **(A)** The variability in progression rates between individuals and across time within individuals arises from the variability in the trend, simulated by $\delta_{t,i}$ and result in the unobserved underlying disease states (gray lines). The observed measurements (i.e., MDS-UPDRS scores, digital assessment scores, etc.) are represented by y_t,i_ in **(A)** and points in **(B)** The vertical distance between the gray line and its associated points represents the variability induced by the measurement process, $\varepsilon_{t,i}$. **(C)** The three main study designs considered: (1) quarterly clinic visits, (2) bursts of 6 assessments 8 times per year, and (3) weekly individual assessments.

We generated simulated study data from a Gaussian state space model of PD progression and measurement (Figure 1) that showed a good fit to longitudinal MDS-UPDRS data from the PPMI cohort (15), see Supplementary Text for further discussion of the modeling framework). In brief, unobserved underlying disease severity, θ, is simulated for a study population of size *n* by randomly drawing *n* initial values from a normal distribution. Each participant's disease severity is updated to the next time step by adding the mean trend, τ, (i.e., the underlying disease progression rate) plus Gaussian noise representing variability in the progression process (σ*_T_*). The rate of disease progression, τ, is the only parameter that differs between placebo and DMT study arms. The updating procedure is repeated for each participant across the length of a simulated trial with Q observed timepoints. Observed values, *y*, are then simulated from the time series of underlying disease severity, θ, by adding normally distributed measurement error, *v*, representing a combination of inter- and intra-rater reliability and short-term fluctuations that are not related to underlying disease progression. The updating process is encapsulated in the following equations, for i∈{1,2,…n} and t∈{1,2,…Q}.

$$y_{t,i}=\theta_{t,i}+v_{t,i},\;v_{t,i}∼N(0,\;\sigma_{m})$$

$$\theta_{t,i}=\theta_{t-1,i}\;+\;w_{t,i},\;w_{t,i}∼N(\tau ,\;\sigma_{T})$$

**Table 1:** Parameters used in main simulations (Figure 2).

Parameter	MDS-UPDRS part III score [values from Evers (15)]	Digital at-home step length (meters)
τ	2.63 year^−1^ (13% year^−1^)	0.04–0.07 (8%–13% year^−1^)
σ_T_	5.58 year^−1^	0.15 year^−1^
σ_m_	3.94^a^	0.06^b^

^a^ Independent estimate from Martinez-Martin (8): 4.3.

^b^ Estimated from mPower data.

The elements of clinical study design included in the simulations were the number of participants per study arm, schedule of assessments, and study duration. For simulations of in-clinic MDS-UPDRS part III scores, all parameters were taken from estimates described in Evers et al. (15).

For simulations of digital at-home measures, measurement parameters were estimated from the mPower data (i.e., starting mean, μ*_s_*, starting standard deviation, σ*_s_* and standard error of the measurement, σ*_m_*, as described above for individual assessments). Bursts were simulated by drawing 6 individual assessments per burst and taking the median. Unfortunately, we lack empirical estimates of the trend and trend variance (τ and σ^2^*_T_*) from at-home assessments because we do not have sufficient longitudinal data on digital measures to disentangle measurement error from progression variability.

Because τ and σ^2^*_T_* represent the trend and trend variance in underlying disease progression, respectively, we began by assuming that these are independent of measurement type and scale with the mean value of a measure, which allowed us to estimate them from the in-clinic measures. That is, τ_digital_ = τ_clinic_ (μ_digital_/μ_clinic_) and σ_T,digital_ = σ_T,clinic_ (μ_digital_/μ_clinic_). However, while in-clinic and at-home assessments both measure underlying motor function, they do so in somewhat different ways, and we therefore relaxed this assumption and considered the robustness of our results to the possibility that at-home measures may be less responsive than in-clinic measures by reducing τ_digital_ to varying degrees relative to in-clinic measures. We modeled the effect, e, of a DMT as a reduction in the progression rate, τ, such that the progression rate in the treatment arm is eτ, where 0 < e ≤ 1.

### 2.4 Study designs

We considered three core study designs (Figure 1C): (1) in-clinic MDS-UPDRS every 3 months, (2) 48 digital at-home assessments per year clustered into 8 bursts of six assessments each, and (3) 48 digital at-home assessments per year evenly spaced across the study duration. We additionally assessed the robustness of our results to study designs with different clustering of bursts by grouping the 48 assessments into 4, 6, 12 and 24 bursts.

### 2.5 Progression rate estimation from simulations & power calculations

We used these simulations to determine the statistical power of a clinical trial to detect treatment effect. Statistical power measures the sensitivity of a study to an effect of interest and is used here to compare the sensitivity of different longitudinal study designs to detect reduced PD progression induced by a DMT. After simulating data for placebo and treatment arms, we assessed the power to detect a treatment effect by fitting a linear mixed effects model to the simulated observations, y, with fixed effects for time, study arm and their interaction, and a random intercept for participant. A first-order autoregressive, AR(1), process was used to model the residual covariance structure between observations within participants following model selection. Model residuals were examined to assess goodness of fit.

For every set of parameters, 1,000 simulations were run and statistical power was calculated as the proportion of assessments for which the coefficient of the interaction term for treatment-arm-by-time had a *p*-value <0.05, as determined from a t-distribution with the appropriate degrees of freedom using the R package *nlme* (24). An additional criterion for trial success is included in the supplement; in these simulations, in addition to a *p*-value <0.05, the mean difference in change between the treatment and placebo group across the study duration must exceed the MDC for the measure of interest.

### 2.6 Software

ICC calculations were performed using Python 3.11 and simulations and power calculations were carried out in R 4.2.1 (25). The code used for analysis and simulations is available upon request.

## 3 Results

### 3.1 Reliability of at-home digital PD assessments

**Figure 2:**
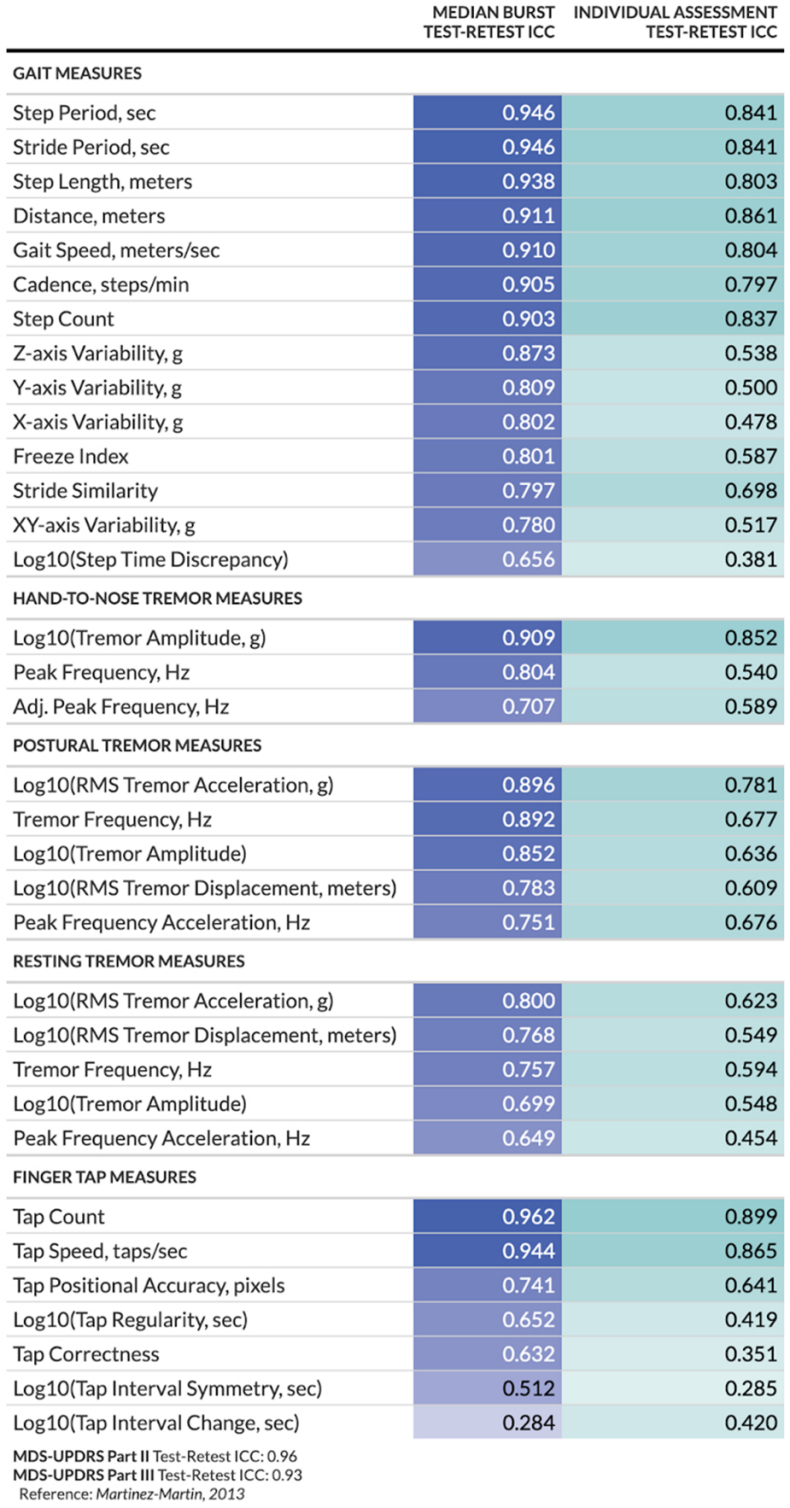
Test-retest reliability per digital at-home measure and study design. For burst assessments, test-retest reliability is calculated between the median of measurements within each burst; for individual assessments, test-retest reliability is calculated between the individual measurements.

We assessed the reliability of at-home digital measures obtained from (1) a 30-s walk test (“gait measures”), (2) a speeded finger tapping assessment (“finger tap measures”), and (3) a tremor assessment (“tremor measures”). Figure 2 summarizes the test-retest reliability as measured by ICCs for each at-home digital measure, separated by whether they were considered individually or an average across multiple measures taken within a 7-day period. Measurements obtained from bursts are summarized by calculating the median value per burst. A median of 6 measurements (mean = 4.6, standard deviation = 2.6) were included in each burst calculation. Most measures obtained from individual or burst assessments exhibited good-to-excellent reliability (26). However, several measures showed poorer reliability overall (e.g., log step time discrepancy, log tap interval symmetry, and tap correctness, collected during individual assessments; log tap interval change collected during burst assessments). The modeling approach used for estimation appeared reasonable based on Q-Q plots and other visualizations of residuals (Supplementary Figure 1). The MDC varied across measures, ranging from less than 10% of the mean (e.g., postural tremor displacement) to over 150% of the mean (e.g., change in tap interval) (Supplementary Tables 2, 3).

### 3.2 Power calculations for at-home measures & study design implications

Power calculations were carried out by fitting a linear mixed model to data generated from the Gaussian state space model. Examination of model residuals suggested a reasonable fit between the model used for effect detection and that used for data generation (Supplementary Figure 2). A comparison between mixed models with and without an autoregressive correlation structure of order 1 AR(1) indicated a significantly better fit by AIC values for the AR(1) model (Supplementary Figure 3), and that model is used for all power calculations presented here.

**Figure 3:**
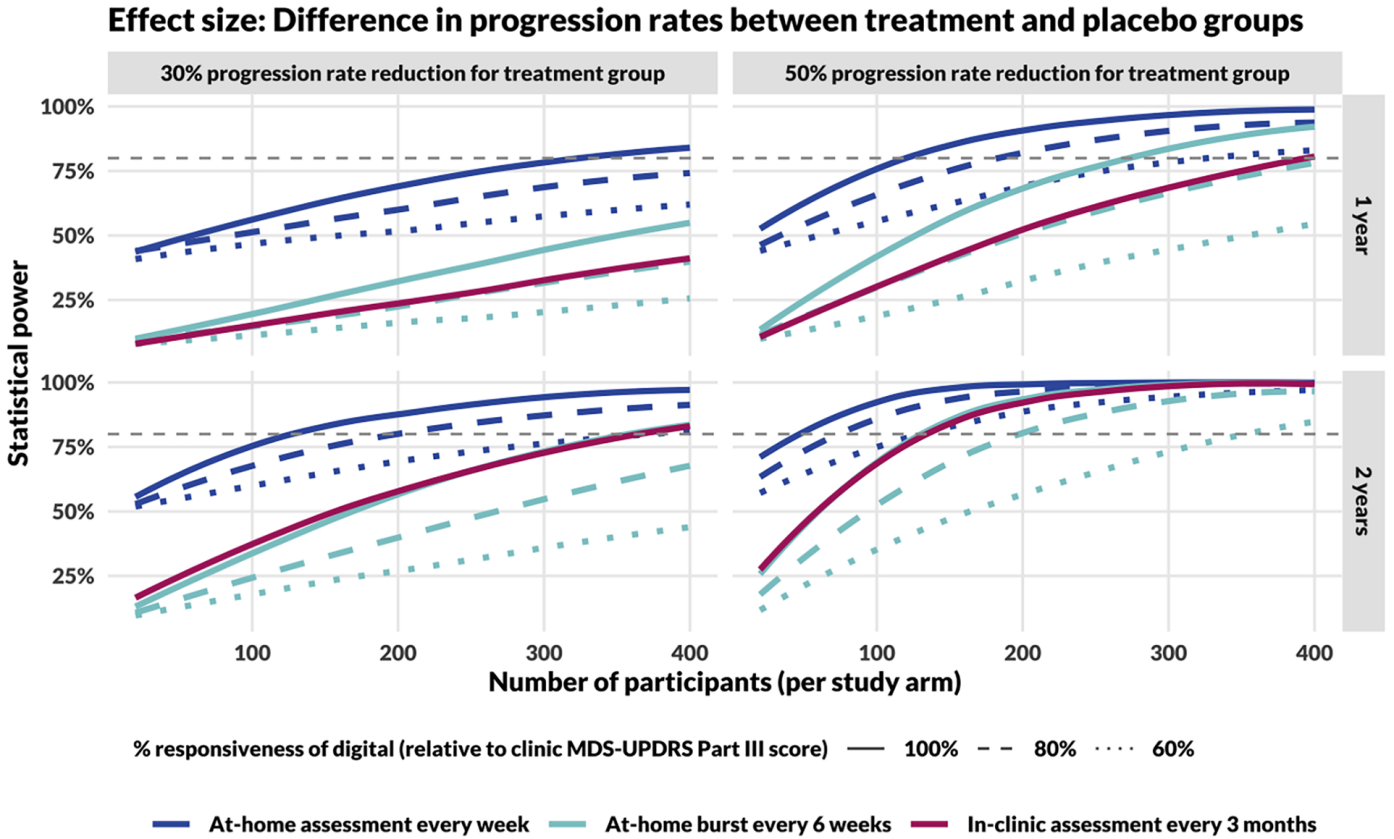
Power curves comparing study designs incorporating in-clinic and at-home assessments. The top row shows results for 1-year long studies, the bottom for 2-year. The left column models a DMT that reduces disease progression rate by 30% and the right column by 50%. In comparing DMT with placebo cohorts, the effect size for calculating study power is the difference in slopes of the measure over time (MDS-UPDRS Part III score for in-clinic assessments, Step Length during the 20-s walk test for at-home assessments), assessed using a linear mixed-effects model. Sample size calculations for in-clinic assessments (red, dashed line) assume responsiveness to progression and measurement error estimated by Evers et al. (15). The gray, dashed line represents the threshold for 80% power.

Based on empirical estimates of measurement error in digital and in-clinic assessments, and assuming that digital measures progress at the same rate as in-clinic measures after rescaling to account for different units, repeated at-home assessments consistently outperformed in-clinic assessments taken once every 3 months, regardless of whether the digital assessments were implemented in bursts or assessed weekly (evenly spaced), during a 1-year trial (Figure 3). For 2-year trials, at-home assessments implemented in bursts perform similarly to in-clinic assessments taken once every 3 months, assuming equivalent responsiveness.

As the responsiveness of digital measures (i.e., the trend, τ) decreases compared with clinic MDS-UPDRS Part III total score, statistical power decreases, regardless of the method of at-home assessment (collected weekly or within bursts). However, for the full range of parameters considered in these simulations, weekly at-home assessments retained higher statistical power compared to in-clinic assessments performed once every 3 months.

Additionally, the temporal spacing of at-home measures had a significant impact on statistical power. Study designs incorporating weekly assessments (48 assessments per year) consistently outperformed designs incorporating at-home bursts every 6 weeks (8 median bursts per year). Further, we found that a more even distribution of assessments always increased power under the assumption that the reliability was the same (Supplementary Figure 4). For example, 48 individual assessments provided greater power than 24 bursts of 2, which provided more power than 12 bursts of 4, and so on.

Based on an 80% statistical power threshold, we can make several different comparisons in sample size requirements between different study designs. For example:

• Assuming a 30% progression rate reduction and 100% responsiveness of the digital measure, a 2-year study would require approximately 110, 350, and 350 participants per study arm based on measures obtained weekly at-home, in 6-week bursts at home, and in-clinic every 3 months, respectively.
• Assuming a 50% progression rate reduction and 100% responsiveness of the digital measure, a 1-year study would require approximately 110, 270, and 390 participants per study arm based on endpoints obtained weekly at-home, in 6-week bursts at home, and in-clinic every 3 months, respectively.

We additionally considered the sensitivity of power calculations to estimates of trend and measurement error. The results indicated that in the presence of high variability in progression rates (σ^2^*_T_* = 30 for MDS-UPDRS part III total score), in the range estimated for PD (15), measurement error had little effect on statistical power (Supplementary Figure 5). In contrast, when progression rates had less variability (e.g., σ^2^*_T_* = 1 or 5), a more precise measure (e.g., σ^2^*_m_* = 1 or 5) substantially increased statistical power, especially for infrequent assessments. An increase in trend error of 20%–40% increases necessary sample sizes (Supplementary Figure 6), but its impact is less than that of a 20%–40% decrease in measure responsiveness (Figure 3).

### 3.3 Responsiveness of at-home measures

**Figure 4:**
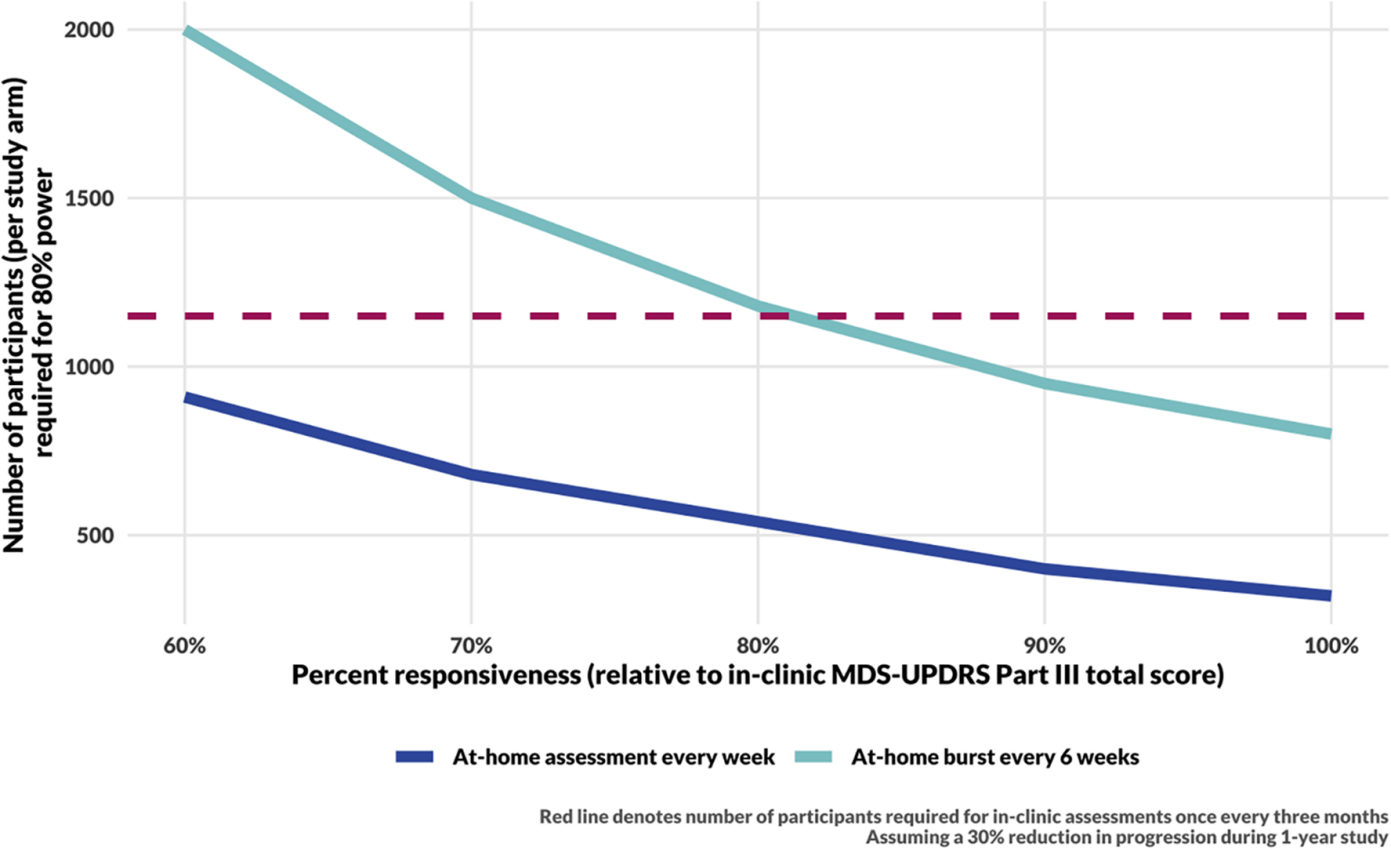
Sample size calculation results for a 1-year long DMT trial. The sample size for in-clinic assessments (red, dashed line) assumes responsiveness to progression and measurement error estimated by Evers et al. (15). Sample size calculations for DHT trials assume measurement error as estimated from the data (see Figure 2, Step Length) and consider a range of responsiveness of digital measures to underlying disease progression. The simulations for the blue curve include 48 assessments per year. The cyan curve includes 8 bursts per year, with 6 assessments per burst.

The responsiveness of digital measures to changes in motor function in PD is not yet well characterized; we therefore consider the impact of reduced responsiveness of a digital measure on the sample size needed for 80% power to detect a 30% reduction in progression rate in a treatment arm throughout a 1-year study (Figure 4). Using at-home assessments taken weekly would allow for detection of a modest 30% reduction in the rate of disease progression within 1 year with fewer than 910 participants per study arm even if the digital assessments were only 60% as responsive to progression as in-clinic MDS-UPDRS Part III total score. In the “ideal” scenario for which digital assessments are as responsive as-clinic MDS-UPDRS Part III, assuming a 30% reduction in the rate of disease progression, such a study would require 320 participants per arm compared to 1,150 per arm in a study that assesses MDS-UPDRS Part III in-clinic every 3 months.

### 3.4 False positive rate and minimum detectable change

Finally, we considered the implications of this modeling approach on the false positive rate. We found that while there was no strong evidence of bias in the estimates (Supplementary Figure 7), the probability of finding a significant difference between study arms when there was none (i.e., type I error) increased with both assessment frequency and trend variance (σ^2^*_T_*, Supplementary Figure 8). One way to manage this is to consider not only statistical but also clinical significance of the results. Indeed, the problem of type I error is mitigated if a simulation is considered to demonstrate study success if and only if the following two criteria are met: (1) the *p*-value for the difference in rates of change between treatment arms is <0.05 and (2) the estimated mean difference in the measure is greater than the minimum detectable change (MDC) (Supplementary Figure 9), though as expected, the probability of study success is reduced in this scenario.

## 4 Discussion

We estimated the reliability of a suite of at-home digital assessments administered on a smartphone to measure motor function in PD and performed simulations of clinical trial designs to assess the ramifications of implementing in-home digital health measures in DMT studies. In agreement with estimates of the reliability of other digital PD measures, we found the test-retest reliability for bursts of digital measures were as good as or better than the reliability of MDS-UPDRS part III scores. Interestingly, even though individual digital assessments typically have poorer test-retest reliability than in-clinic or at-home burst assessments, we found that a study design with evenly spaced digital weekly assessments outperformed both alternatives. This result suggests that the key challenge in measuring PD progression stems not from a lack of sufficiently sensitive and reliable measurement tools, but rather from the inherent variability in PD disease burden at points in time that renders infrequent measurement insufficient.

The result of superiority of frequent at-home assessments to in-clinic assessments every 3 months is robust to substantially decreased responsiveness of digital at-home measures compared with in-clinic (Figure 3). However, the quantitative results regarding the necessary sample size were greatly affected by the responsiveness, and this will be important in future trial design. This is a difficult parameter to estimate as it requires longitudinal data. Ongoing and future multi-year studies that incorporate frequent digital measures in PD will be necessary to quantify this [e.g., (6, 27, 28)].

The results of this study suggest that evenly spaced assessments provide greater power than any configuration of an equal number of assessments distributed in bursts. This may be understood in the context of information theory; when compressing data using a logically irreversible process, such as summarizing a burst of assessments with a median, there is inherent loss of information as measured, for example, by Shannon entropy (29). The superiority of evenly spaced assessments also has implications for the implementation of DHTs in clinical trials. Frequent, evenly spaced measures require participants to consistently perform digital assessments across long periods of time. Adherence to at-home assessment regimens in clinical trials may decrease over time [e.g., (30)], and methods for maintaining usage will be important. Additionally, understanding the causes and impacts of missing assessments will be important.

We note that the results assumed progression rates and variability estimated in a patient population on standard of care medications such as levodopa (15). DMT studies are often longitudinal and conducted in patients in the early stages of PD [e.g., (31, 32)], which can include treatment-naive participants. Smaller sample sizes may be sufficient to detect DMT effects in treatment naive individuals, in part because the estimated progression rate is higher in the absence of medication (2). However, while participants may be unmedicated at the start of the study, over the course of a year or more they are likely to start symptomatic treatment (33). This transition can be challenging to account for in models of disease progression, and whether inclusion of covariates such as levodopa equivalent daily dose (LEDD) is sufficient to account for the changes induced by starting treatment remains an open question. There is substantial evidence that digital measures can detect levodopa effects [e.g., (20, 34, 35)], but as of yet, little evidence of detecting progression (14). Further work is needed to identify what clinical variables will be necessary to disentangle temporary fluctuations from underlying disease progression.

A key assumption in this study is that progression in both the treatment and placebo groups, while highly variable, is on average linear with time. Varying rates of progression with time could occur due to intrinsic characteristics of the motor function being measured, a learning effect, or time-dependent treatment effects of a DMT. Prior studies provide evidence for two of these: linear models in time are suitable for some but not all digital measures (14), and learning effects can be detected in at-home measures [e.g., (36, 37)]. As there are no approved DMTs for PD, the importance of time-dependent treatment effects remains unknown, but it is considered in other similar modeling assumptions (38) and is likely relevant. For measures whose progression cannot be approximated as linear, a study design that facilitates treating time as a discrete variable, such as bursts of assessments, may be beneficial. It should also be noted that this study does not model subpopulations within PD that may have different mean progression rates (16). Further work is necessary to understand how this type of heterogeneity in a population may affect the benefits and study design of digital at-home assessments. Additionally, data collected at higher frequency can require consideration of autocorrelation and temporal confounders (39).

One drawback of the mixed effects modeling approach taken in this study for power calculations is the possibility for false positive results. While estimates of trend using linear mixed effect models are largely insensitive to model misspecification (40), the standard error of the fixed effects may be underestimated in the presence of misspecified random effects such as autocorrelation (41, 42). The increased false positive rate with frequent sampling observed in the simulations can be understood in the context of the mismatch between the data generation process (i.e., a random walk with trend) and the model fitting procedure. As described here, one solution to this problem is to require not only statistical but also clinical significance. However, this comes with a loss of power to detect small changes, especially in shorter time windows. Analysis methods tailored to data that arise from underlying processes with this type of autocorrelation may be important in this context (43).

Future work to better understand the biological mechanisms underlying the progression of motor symptoms in PD can inform choices of models used for detecting treatment effects. In this study, the data generating process was chosen because it has been shown to parsimoniously explain heterogeneity present in the disease's dynamics in PPMI data (15), and therefore seems a reasonable candidate for a mechanistic model. The model assumes the mean underlying progression rate is the same across all patients, which we know to be an oversimplification. For example, certain genotypes progress more quickly than others [e.g., (44)]. One outcome of this assumption is that the trend variance reported by Evers (15) may be an overestimate as it accounts for not only random variation across time but also consistent variation between individuals that exists among the PPMI patients.

The mixed model framework used for effect detection has been used in longitudinal assessments of PD progression, including in PPMI data (2). However, while the model may appear to be a reasonable fit based on standard examination of residuals, our results suggest that care needs to be taken to avoid overconfidence in detection of small effects. Given the trade-off between power to detect treatment effect and the false positive rate that results from fitting misspecified models, future work to investigate the underlying mechanisms of motor function progression and the empirical autocorrelation structure of PD measurements will be important. Digital measures may provide an important window into the nuances of PD progression and its variability and allow for empirical examination of temporal correlation structures in data that can help determine optimal analysis methods (20). Given the high and increasing burden of PD around the globe, therapies that can stop or slow its progression will benefit millions of people (45). As of 2023, there were 63 ongoing clinical trials for PD DMTs, including 32 phase II and 6 phase III (46). For these trials to be successful, in addition to an effective therapeutic agent, they must utilize measurements that allow for detection of treatment effect in the face of the high degree of variability inherent to PD progression. This study demonstrates that frequent measures enabled by digital health technologies that can be used consistently in patients’ homes may increase the power to detect treatment effects in smaller and shorter trials.

## Data Availability

Publicly available datasets were analyzed in this study. This data can be found here: [doi: 10.7303/syn4993293].
