# Supplementary material for: Impacts on study design when implementing digital measures in Parkinson's disease-modifying therapy trials
Source: Front Digit Health. 2024 Oct 9;6:1430994. doi: 10.3389/fdgth.2024.1430994 (PMC11496294; doi:10.3389/fdgth.2024.1430994)
Supplement: Supplementary file 1 [file Datasheet1.pdf]

Supplementary Material

Impacts on study design when implementing digital measures in Parkinson's disease-modifying therapy trials

Jennie S Lavine[1*], Anthony D Scotina[1], Seth Haney[1], Jessie P Bakker[1], Elena S Izmailova[1], Larsson Omberg[1*]

[1]Koneksa Health, New York, NY, United States

*** Correspondence:**
Corresponding Authors
jennie.lavine@koneksahealth.com; larsson.omberg@koneksahealth.com

Supplementary Text

Choice of Gaussian state space model for data generation

Parkinson's Disease (PD) progression data was simulated using a Gaussian state space model for two primary reasons:

(1) It is the only model we know of that is strongly motivated in the context of Parkinson's data, fit to a large longitudinal dataset on disease severity, provides evidence of a reasonably good fit to real world data, and recapitulates patterns of regression to the mean (Evers 2019). Holden et al (2018) also fit a progression model to the longitudinal PPMI data; however, while their modeling approach is straightforward and sensible, they provide little motivation for their modeling choices (linear mixed models with unstructured covariance) and little evidence of goodness of fit. Additionally, the model is not well enough described for use in data generation.

(2) The model separates variability in the disease progression process ('process noise') from variability that arises from measurement ('measurement error'). Given the current state of available data on digital measures in PD, we can empirically estimate measurement error despite the lack of large longitudinal datasets on digital disease progression. The state space modeling framework allows us to harness the knowledge gained from longitudinal datasets using MDS-UPDRS to estimate underlying disease progression and overlay the estimates of measurement reliability and error gained from shorter duration studies utilizing digital measures.

To simulate at-home digital data, we needed to modify the model to account for the different scales of digital and in-clinic measures. In moving from in-clinic to digital at-home data, we used the following principles:

1. Measurement error is solely dependent on measurement type and can be estimated from short-term datasets. In support of the idea that measurement error estimated from short term datasets is indeed applicable to the state space model, Martinez-Martin (2012) estimate measurement error from a test-retest reliability on MDS-UPDRS part III off state study and to be 4.31, which is within the confidence interval for the measurement error estimate in Evers et al (CI: [3.49, 4.39]).
2. The rate and variability of disease progression (i.e., the trend and trend variance parameters in the state space model) are independent of measurement type and therefore, once scaled, can be applied to in-clinic or at-home measures.
3. The mean value of a measure provides a reasonable approximation of its scale. We convert the rate and variance of disease progression as measured by in-clinic MDS-UDPRS to at-home digital measures by scaling the in-clinic values by the ratio of the in-clinic to at-home means.

We assess the sensitivity of the power results to assumption (2) and (3) above by considering at-home measures that are 'worse' (i.e., lower trend and higher trend variance) than in-clinic. Main text figure 3 includes dotted and dashed lines for at-home digital trend parameters that are less sensitive to change in underlying disease than in-clinic measures, i.e., the at-home digital trend is

lower than it would be if it scaled with the mean. Supplementary figure XXX shows the same relationship but assuming that trend variance associated with at-home measures is greater than it would be if it scaled with the mean.

Evers, L. J. W., J. H. Krijthe, M. J. Meinders, B. R. Bloem, and T. M. Heskes. 2019. 'Measuring Parkinson's disease over time: The real-world within-subject reliability of the MDS-UPDRS', Mov Disord, 34: 1480-87.

Holden, S. K., T. Finseth, S. H. Sillau, and B. D. Berman. 2018. 'Progression of MDS-UPDRS Scores Over Five Years in De Novo Parkinson Disease from the Parkinson's Progression Markers Initiative Cohort', Mov Disord Clin Pract, 5: 47-53.

Martinez-Martin, P., C. Rodriguez-Blazquez, M. Alvarez-Sanchez, T. Arakaki, A. Bergareche-Yarza, A. Chade, N. Garretto, O. Gershanik, M. M. Kurtis, J. C. Martinez-Castrillo, A. Mendoza-Rodriguez, H. P. Moore, M. Rodriguez-Violante, C. Singer, B. C. Tilley, J. Huang, G. T. Stebbins, and C. G. Goetz. 2013. 'Expanded and independent validation of the Movement Disorder Society-Unified Parkinson's Disease Rating Scale (MDS-UPDRS)', J Neurol, 260: 228-36.

Supplementary Figures

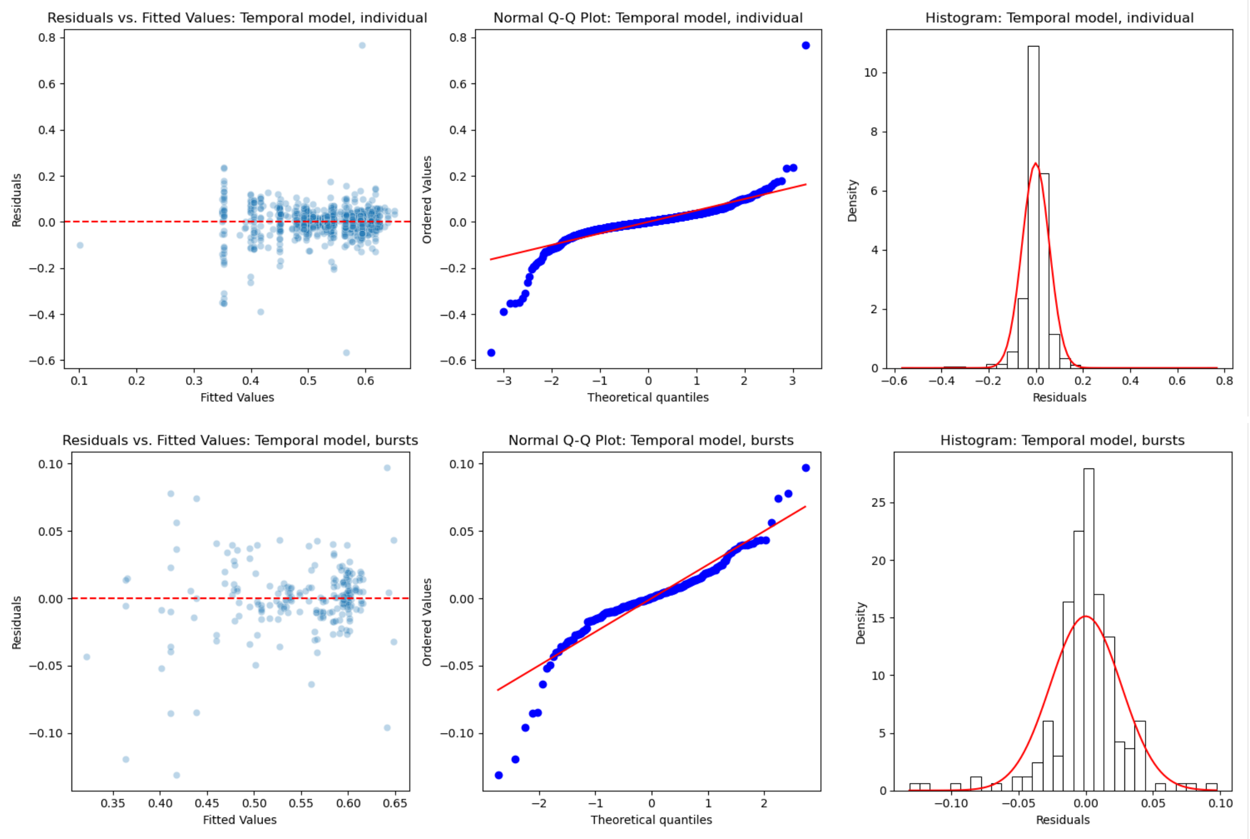

Supplementary Figure 1. Model residuals from the fit of the linear random-intercept model used to compute ICCs and MDCs for the measure of step length.

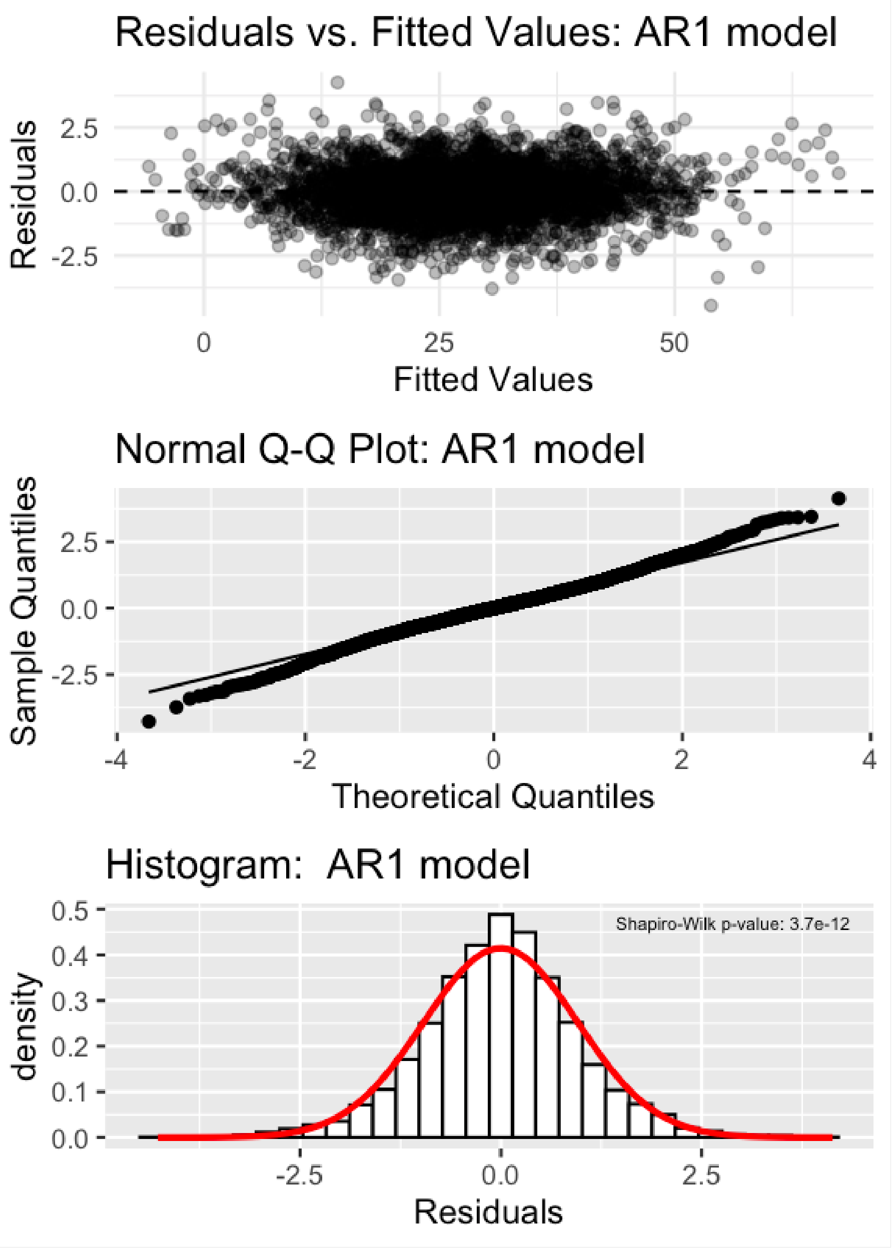

Supplementary Figure 2. Model residuals from 100 simulations using the parameters described in Main Figure 1. The Q-Q plot shows some evidence of fat tails, but overall, the residuals suggest a reasonably good fit between the linear model used for estimation and the data generated by the Gaussian state space model.

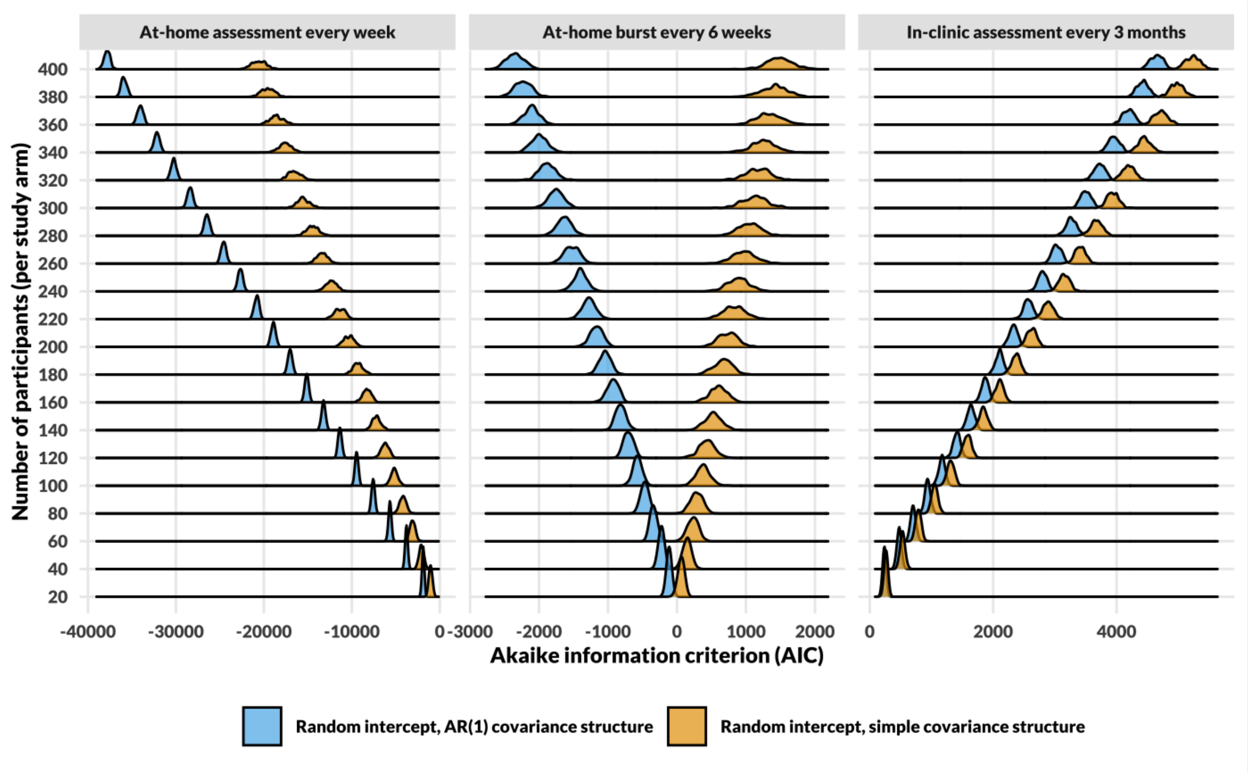

Supplementary Figure 3:

Distributions of AIC values from 400 simulations per study configuration using a mixed effects model with (blue) or without (tan) autocorrelated residuals. Each study configuration assumes a 1-year study duration, 100% responsiveness of the digital measure relative to in-clinic MDS-UPDRS Part III score, and a 50% progression reduction in the treatment group. The AR(1) model is consistently a better fit (i.e., lower AIC value) across all study configurations.

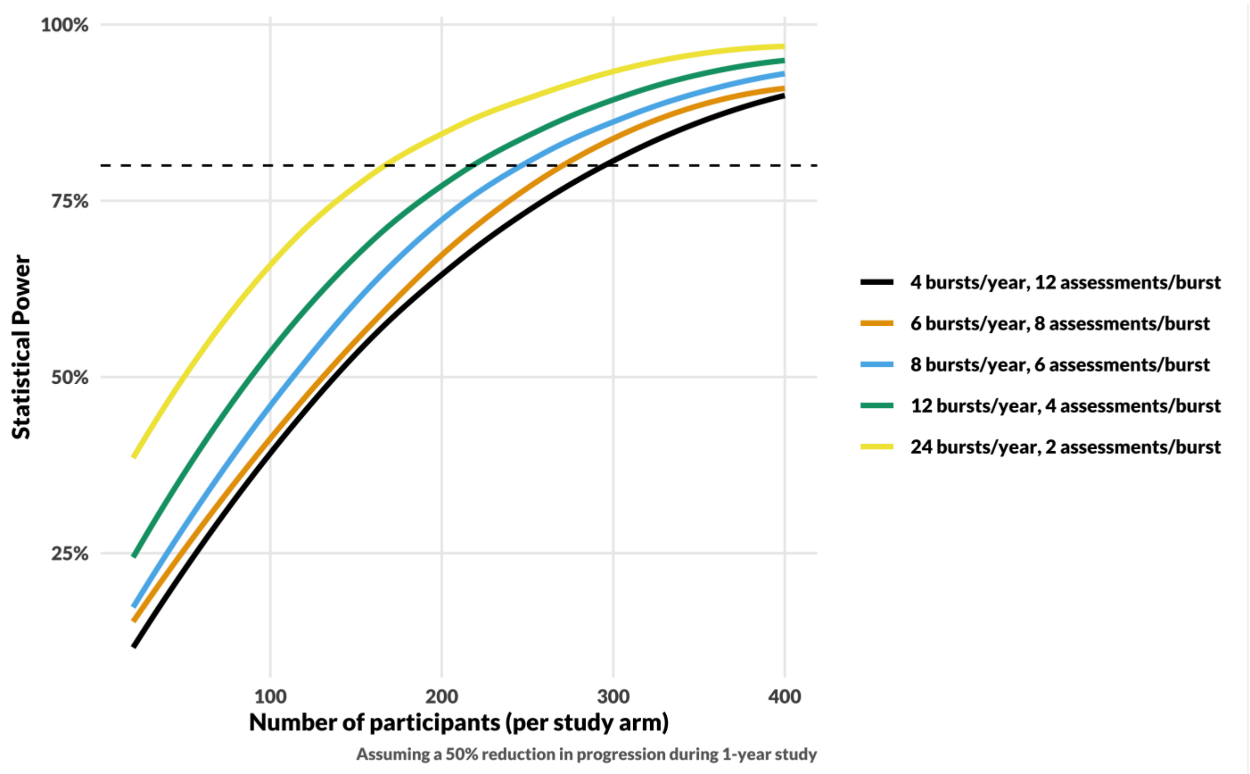

Supplementary Figure 4: Power curves for schedules of assessments with different distributions of 48 measures within bursts. The most even distribution (yellow curve, 24 bursts of 2 assessments each) has the highest power and the most clustered (black, 4 bursts of 12 assessments) has the lowest power. Parameters for trend, trend variance and measurement error were set to MDS-UPDRS part III values (see table 1). The simulated year-long study included a DMT that reduces progression rate by 50%.

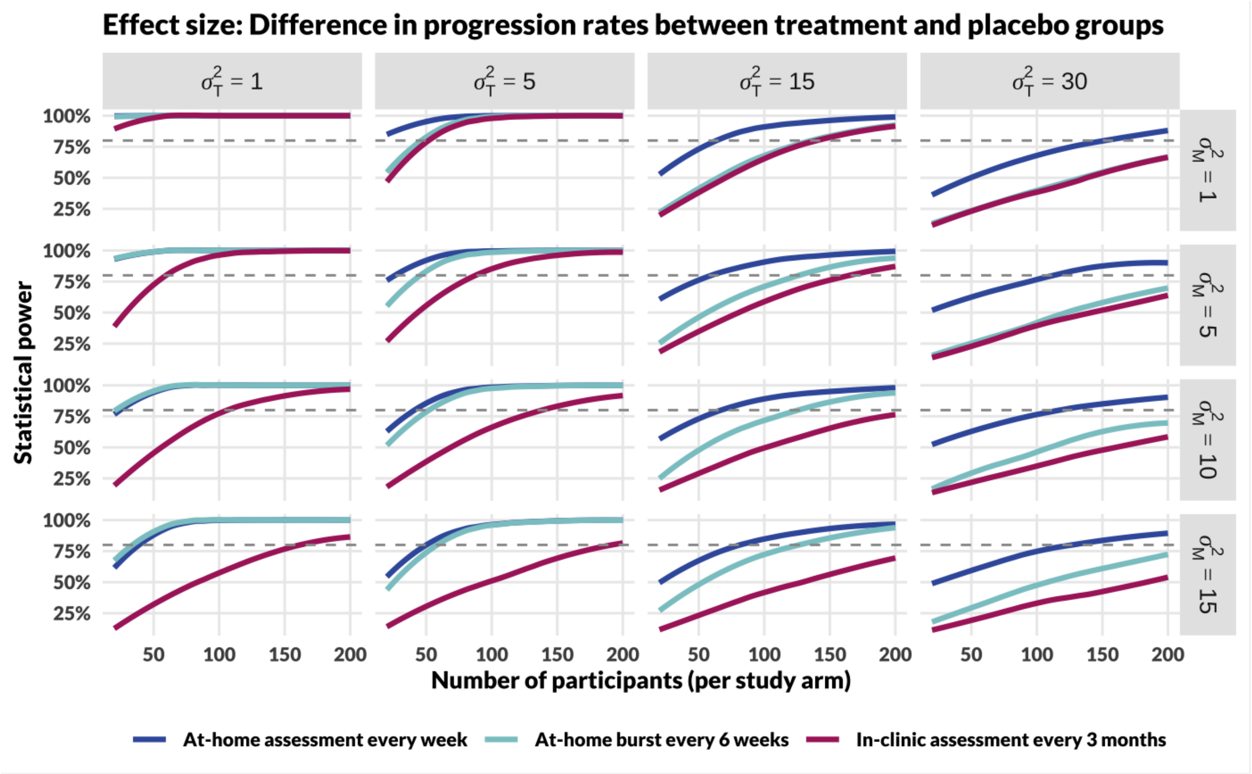

Supplementary Figure 5. Power curves comparing different measurement errors and trend variances between study designs incorporating in-clinic and at-home assessments. In comparing DMT with placebo cohorts, the effect size for calculating study power is the difference in slopes of MDS-UPDRS Part III score over time, assessed using a linear mixed-effects model. Sample size calculations assume a 1-year study design, equal responsiveness to progression between designs, and a 50% progression rate reduction for the DMT cohort.

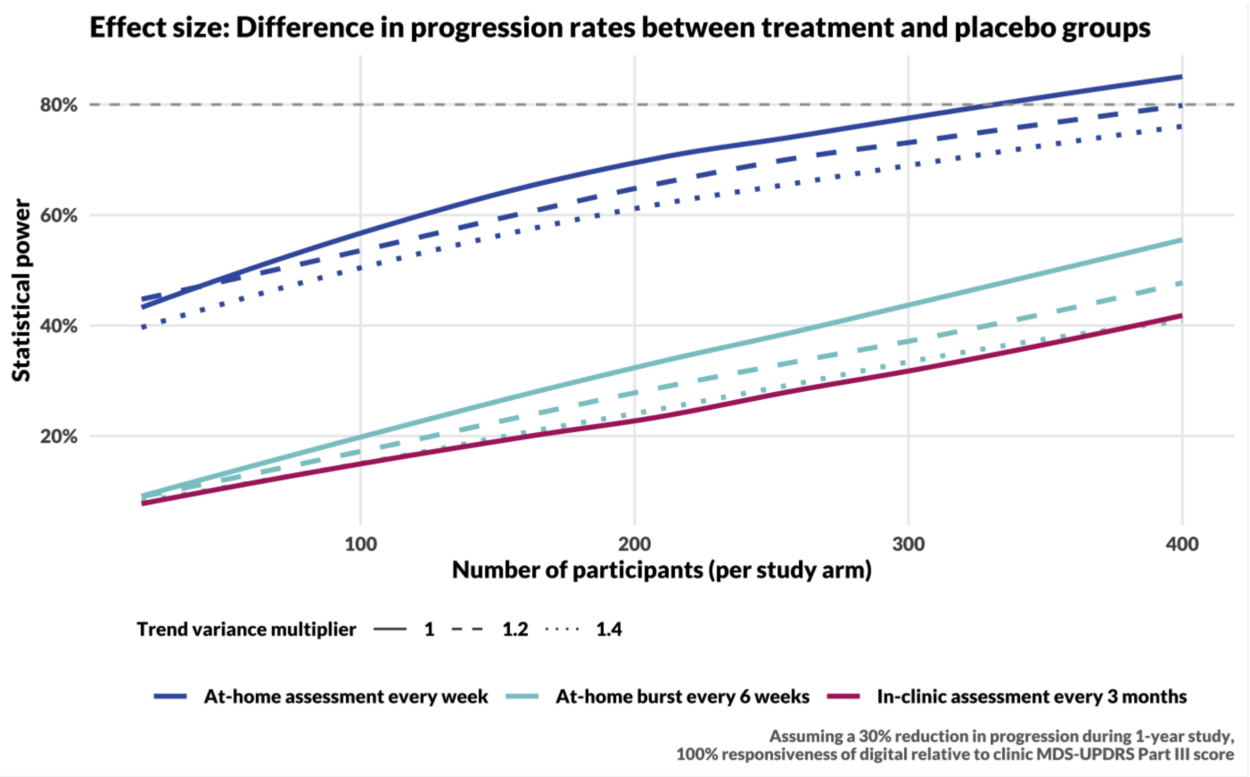

Supplementary Figure 6: Power calculations showing the impact of digital measures with trend variance that does not scale with its mean compared with in-clinic measures. Parameters are the same as those used in Fig 3 upper left panel (i.e., a 1-year study with a DMT that reduces the disease progression rate by 30%) except for the trend variance, which is increased for the dashed and dotted lines by 20% and 40%, respectively, over a trend variance that scales with its mean.

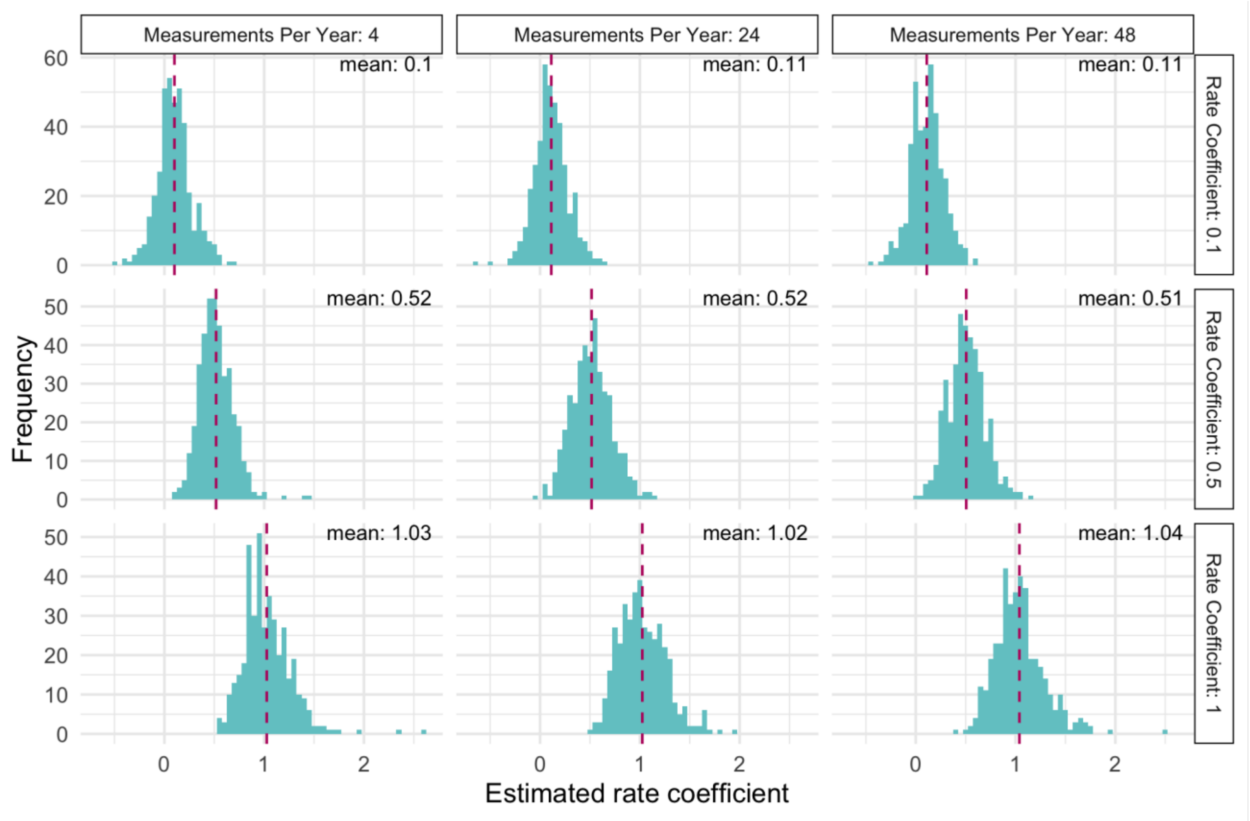

Supplementary Figure 7: Distributions of estimates of the rate coefficient for different simulation configurations. The rows indicate treatments ranging from 90% effective (top row, rate coefficient = 0.1) to completely ineffective (bottom row, rate coefficient = 1), and across different numbers of assessments spread throughout the year (i.e., 4, 24, and 48). The estimates of the difference between the treatment and placebo group are centered near the true value (i.e., the values on the right-hand side).

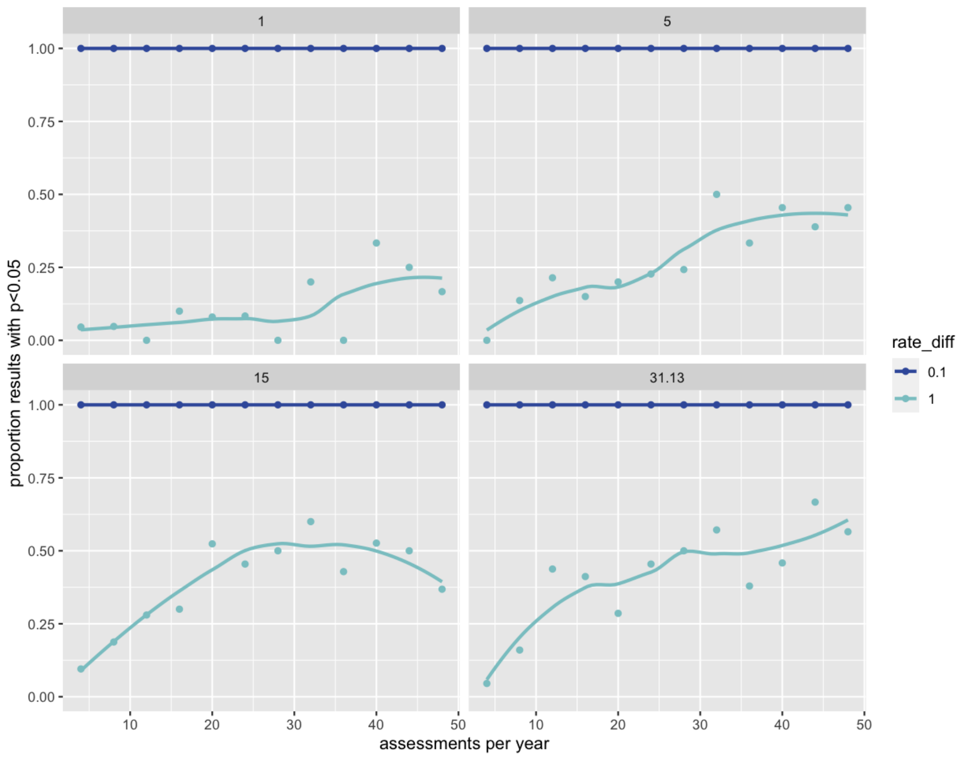

Supplementary Figure 8: The panels show 4 different levels of trend variance, ranging from 1 to 31.13. The cyan lines indicate type I error; the true drug effect in these simulations was 0. For low trend variance (upper left), there is low type I error across assessment frequency (x-axis). As trend variance increases, type I error increases. In the presence of moderate or high trend variance (e.g. trend variance =15 or 31.13), type I error increases with increasing frequency of assessments. Results shown are for a 2-year long study with 48 weekly assessments with characteristics similar to MDS-UPDRS part III (i.e., σ^2_m = 15.52, τ=3).

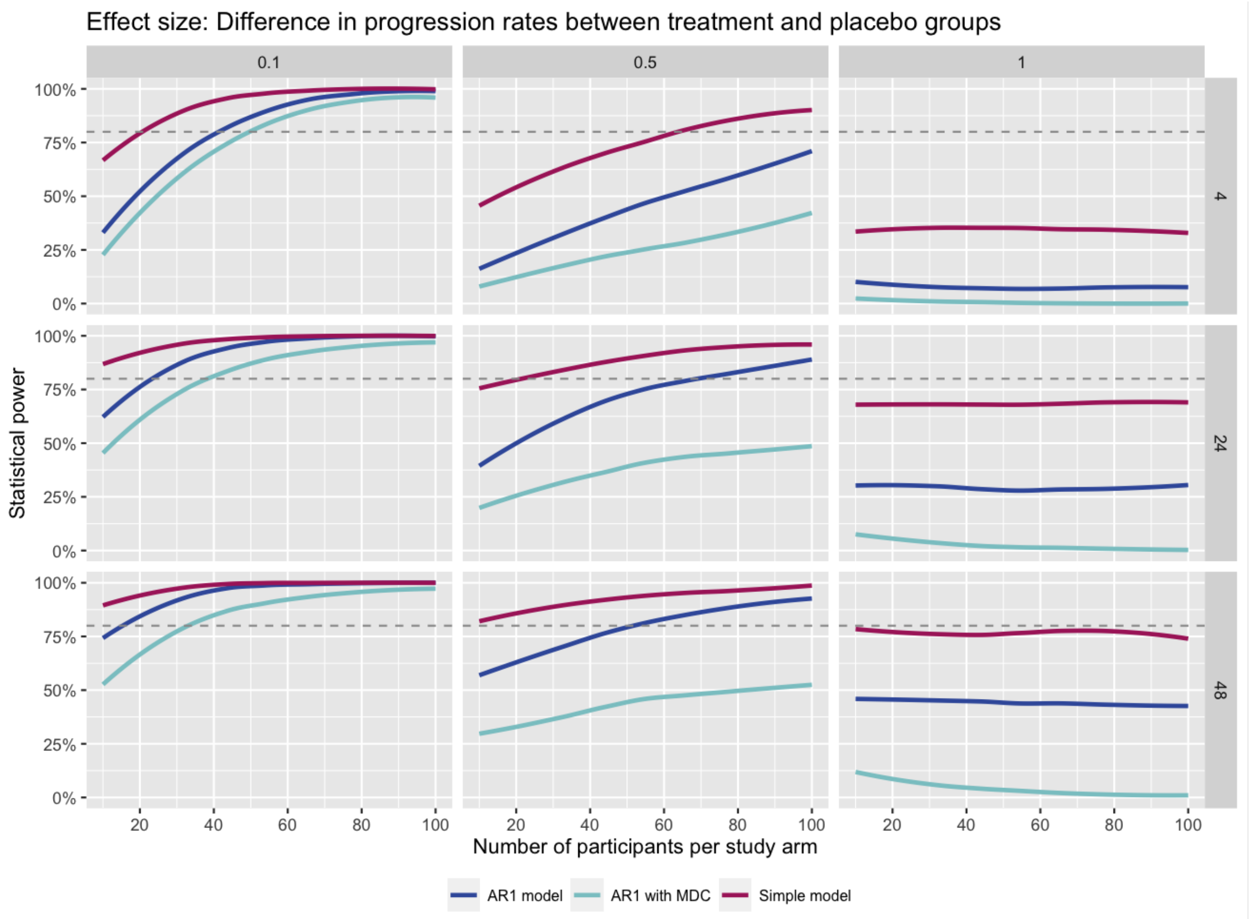

Supplementary Figure 9: Impact of MDC criterion for success. Cyan lines show power curves when a successful trial is defined by $p<0.05$ for the interaction term in an AR1 model and the mean difference in change across 2 years > MDC, which greatly reduces type I error (right-hand column, magenta line for simple model indicate high type I error, blue line AR1 shows type I error reduction, and cyan line for AR1 model with MDC criterion shows type I error mitigation). The inclusion of the criteria also reduces power to detect a difference when it is present (columns 1 through 3). The type I error is most pronounced for trials with very frequent assessments (bottom row, 48 assessments per year), but even in this scenario, inclusion of the MDC criteria mitigates the problem with type I error. The results are shown for a 2-year long study using the digital measure of stride period ($\sigma^2_m = 0.011$, $\sigma^2_T = 0.105$, $\tau = 0.152$).

Supplementary Tables

Supplementary Table 1. Survey of digital tools in PD.

Citation	Assessment	Measure	Aggregation	N	ICC	Notes
Burq .et al 2022	Tremor	Rest tremor lateral acceleration	Aggregate (monthly)	138	0.96	Sensitive to ON/OFF fluctuations (Cohen's d = 0.19-0.54). Moderate to strong correlations with UPDRS part III for tremor (r=0.7) and gait (r=-0.46).
		Rest tremor lateral amplitude			0.94	
		Rest tremor total acceleration			0.95	
		Postural tremor lateral acceleration			0.94	
		Postural tremor lateral amplitude			0.94	
		Postural tremor total acceleration			0.93	
	Gait	Arm swing acceleration		139	0.75	
		Peak arm swing energy			0.82	
		Arm swing foward acceleration			0.89	
		Cadence			0.76	
Sahandi Far .et al 2021	Gait	Number of steps	Individual	1417	0.25	Notable for largest number of subjects. Mpower data.
		Freeze index z			0.26	
		Mean stride interval			0.29	
	Tap	Number of taps		2644	0.64	
		Range tap interval			0.27	
Jakob .et al 2021	Gait	Gait speed	Individual	50	0.99	
		Stride length			0.99	
		Stride time			0.97	
Lipsmeier . et al 2018	Tremor	Rest tremor skewness	Aggregate (2 weeks)	78	0.9	Digital features significantly differentiate HC from PD subjects (p<0.001 for both tremor and tap).Shows a significant correlation between feature and aligned UPDRS item (p<0.05 for both tremor and tap).
		Postural power			0.97	
	Tap	Tap variability			0.64	
Lipsmeier . et al 2022	Tap	Tap variability (most affected side)	Aggregate (2 weeks)	300	0.88	All tap and tremor features correlated significantly with aligned UPDRS items (p<1e-4). Used as exploratory endpoint in PASEDENA clinical trial.
		Tap variability (least affected side)		300	0.84	
	Tremor	Rest tremor energy (most affected side)		300	0.94	
		Rest tremor energy (least affected side)		300	0.91	
		Postural tremor energy (most affected side)		300	0.92	

		Postural tremor (least affected side)		300	0.83	
Makai-Boloni .et al 2021	Tap	Number of taps (two finger tap)	Individual	14	0.94	Notable for direct comparison of one and two finger tap. One finger tap involves tapping alternative sides of a large tablet.
		Tap accuracy (two finger tap)			0.81	
		Inter tap interval SD (two finger tap)			0.86	
		Number of taps (one finger tap)	Individual		0.86	
		Tap accuracy (one finger tap)			0.41	
		Inter tap interval SD (one finger tap)			0.2	
Oyama .et al 2023	Tremor	Postural tremor amplitude	Individual	58	0.04	Direct comparisons are possible bewteen individual (day-to-day) ICCs and aggregate (week-to-week) ICCs.
		Postural tremor acceleration		58	0.46	
		Rest tremor amplitude		62	0.24	
		Rest tremor acceleration		62	0.39	
		Postural tremor amplitude	Aggregate (1 week)	58	0.09	
		Postural tremor acceleration		58	0.55	
		Rest tremor amplitude		62	0.62	
		Rest tremor acceleration		62	0.63	
	Gait	Cadence	Individual	56	0.52	
		Arm swing		56	0.55	
		Cadence	Aggregate (1 week)	56	0.7	
		Arm swing		56	0.73	
Wissel .et al 2017	Tap	Total taps	Individual	11	0.96	Number of taps, tap interval, and tap accuracy could significantly differentiate ON vs OFF state. ICCs are from OFF state

Table 1 References

Burq M, Rainaldi E, Ho KC, Chen C, Bloem BR, Evers LJW, et al. Virtual exam for Parkinson's disease enables frequent and reliable remote measurements of motor function. NPJ Digit Med. (2022) 5:65. doi: 10.1038/s41746-022-00607-8

Jakob V, Kuderle A, Kluge F, Klucken J, Eskofier BM, Winkler J, et al. Validation of a Sensor-Based Gait Analysis System with a Gold-Standard Motion Capture System in Patients with Parkinson's Disease. Sensors (Basel). 2021;21(22). doi: 10.3390/s21227680

Lipsmeier F, Taylor KI, Kilchenmann T, Wolf D, Scotland A, Schjodt-Eriksen J, et al. Evaluation of smartphone-based testing to generate exploratory outcome measures in a phase 1 Parkinson's disease clinical trial. Mov Disord. 2018;33(8):1287-97. doi: 10.1002/mds.27376

Lipsmeier F, Taylor KI, Postuma RB, Volkova-Volkmar E, Kilchenmann T, Mollenhauer B, et al. Reliability and validity of the Roche PD Mobile Application for remote monitoring of early Parkinson's disease. Sci Rep. 2022;12(1):12081. doi: 10.1038/s41598-022-15874-4

Makai-Boloni S, Thijssen E, van Brummelen EMJ, Groeneveld GJ, Doll RJ. Touchscreen-based finger tapping: Repeatability and configuration effects on tapping performance. PLoS One. 2021;16(12):e0260783. doi: 10.1371/journal.pone.0260783

Oyama G, Burq M, Hatano T, Marks WJ Jr, Kapur R, Fernandez J, et al. Analytical and clinical validity of wearable, multi-sensor technology for assessment of motor function in patients with Parkinson's disease in Japan. Sci Rep. (2023) 13:3600. doi: 10.1038/s41598-023-29382-6

Sahandi Far M, Eickhoff SB, Goni M, Dukart J. Exploring test-retest reliability and longitudinal stability of digital biomarkers for Parkinson disease in the m-Power data set: cohort study. J Med Internet Res. (2021) 23:e26608. doi: 10.2196/26608

Wissel, BD, Mitsi, G, Dwivedi, AK, Papapetropoulos, S, Larkin, S, Lopez Castellanos, JR, et al. (2017). Tablet-Based Application for Objective Measurement of Motor Fluctuations in Parkinson Disease. Digit Biomark, 1(2), 126-135. https://doi.org/10.1159/000485468

Supplementary Table 2: Measure definitions. RMS refers to “root mean square”

Measure type	Measure, units	Definition
Gait	Cadence, steps/min	Number of steps taken per minute
Gait	Distance, meters	Distance covered during the walking period
Gait	Freeze Index, N/A	A measure of whether freezing-of-gait occurred
Gait	Gait Speed, meters/sec	Gait speed per walking period
Gait	Log10(Step Time Discrepancy, N/A)	Log-transformed measure of stride step variability (i.e., the symmetry between left and right steps); ranges from 0 to 0.5
Gait	Step Count, count	Number of steps taken during the walking period
Gait	Step Length, meters	Average step length during walking period
Gait	Stride Period, sec	Time between one foot striking the floor and the same foot striking the floor a second time measured in seconds, averaged over the walking period
Gait	Step Period, sec	Half of stride period
Gait	Stride Similarity, N/A	Measure of how similar the strides are to each other within the walking period in terms of overall acceleration profile over the duration of a stride; ranges from 0 to 1
Gait	XY-axis Variability, g	Variability in acceleration in the horizontal plane
Gait	X-axis Variability, g	Variability in acceleration along the axis perpendicular to direction of travel
Gait	Y-axis Variability, g	Variability in acceleration along the axis in the direction of travel
Gait	Z-axis Variability, g	Variability in vertical acceleration
Hand-to-nose tremor	Log10(Tremor Amplitude, g)	90th percentile of the band passed filtered tremor magnitude signal.
Hand-to-nose tremor	Peak Frequency, Hz	Frequency with maximal power in the spectrum formed by adding x,y,z component spectra.
Hand-to-nose tremor	Adj. Peak Frequency, Hz	Frequency with maximal power in the spectrum formed by adding x,y,z component spectra after adjusting for 1/f power decay.
Postural/Resting tremor	Log10(RMS Tremor Acceleration, g)	Log-transformed measure of the acceleration within the range of 2 to 10 Hz
Postural/Resting tremor	Log10(RMS Tremor Displacement, meters)	A measure of the displacement of the assessment phone within the range of 2 to 10 Hz
Postural/Resting tremor	Log10(Tremor Amplitude, N/A)	Log-transformed measure of the maximal normalized power within the range of 2 to 10 Hz
Postural/Resting tremor	Peak Frequency Acceleration, Hz	Frequency within the range of 2 to 10 Hz with maximal power in the spectrum formed by adding x,y,z component spectra.
Postural/Resting tremor	Tremor Frequency, Hz	Frequency within the range of 2 to 10 Hz with maximal power in the tremor magnitude signal.
Finger tap	Log10(Tap Interval Change)	Log-transformed measure of the change in tapping speed over the course of the assessment
Finger tap	Log10(Tap Interval Symmetry, sec)	Log-transformed measure of the absolute difference between (i) the mean time between a left tap and subsequent right tap, and (ii) the mean time between a right tap and subsequent left tap

**Finger tap**	**Log10(Tap Regularity, sec)**	Log-transformed measure of the variability in the time interval between successive taps
**Finger tap**	**Tap Correctness, N/A**	Ratio of the number of the correct button tapped over the total number of taps
**Finger tap**	**Tap Count, count**	Number of taps in an assessment
**Finger tap**	**Tap Positional Accuracy, N/A**	A measure of the positional accuracy of each tap
**Finger tap**	**Tap Speed, taps/sec**	Average number of taps per second

Supplementary Table 3: Minimum detectable change (MDC) per measure, for study designs incorporating burst or individual assessments.

Measure type	Measure	MDC (Burst assessments)	MDC (Individual assessments)	Mean	Ratio (MDC burst / mean)
**Gait**	**Cadence**	14.155	21.011	103.877	0.136
**Gait**	**Distance**	7.937	9.97	26.879	0.295
**Gait**	**Freeze Index**	0.718	1.122	5.157	0.139
**Gait**	**Gait Speed**	0.247	0.368	0.963	0.256
**Gait**	**Log10(Step Time Discrepancy)**	1.115	1.785	-2.426	0.460
**Gait**	**Step Count**	9.556	12.591	48.212	0.198
**Gait**	**Step Length**	0.095	0.165	0.547	0.174
**Gait**	**Step Period**	0.082	0.143	0.590	0.139
**Gait**	**Stride Period**	0.165	0.285	1.180	0.140
**Gait**	**Stride Similarity**	0.228	0.282	0.637	0.358
**Gait**	**XY-axis Variability**	0.045	0.074	0.094	0.479
**Gait**	**X-axis Variability**	0.038	0.069	0.094	0.404
**Gait**	**Y-axis Variability**	0.064	0.115	0.160	0.400
**Gait**	**Z-axis Variability**	0.054	0.107	0.132	0.409
**Hand-to-nose tremor**	**Log10(Tremor Amplitude)**	0.18	0.232	-0.599	0.301
**Hand-to-nose tremor**	**Peak Frequency**	2.103	3.527	6.436	0.327
**Hand-to-nose tremor**	**Adj. Peak Frequency**	2.388	3.035	7.250	0.329
**Postural tremor**	**Log10(RMS Tremor Acceleration)**	0.233	0.357	-1.826	0.128
**Postural tremor**	**Log10(RMS Tremor Displacement)**	0.267	0.407	-3.672	0.073
**Postural tremor**	**Log10(Tremor Amplitude)**	0.246	0.436	-2.242	0.110
**Postural tremor**	**Peak Frequency Acceleration**	3.093	3.486	6.103	0.507
**Postural tremor**	**Tremor Frequency**	2.377	4.39	4.555	0.522
**Resting tremor**	**Log10(RMS Tremor Acceleration)**	0.377	0.575	-2.117	0.178
**Resting tremor**	**Log10(RMS Tremor Displacement)**	0.416	0.679	-4.046	0.103

**Resting tremor**	**Log10(Tremor Amplitude)**	0.264	0.373	-2.515	0.105
**Resting tremor**	**Peak Frequency Acceleration**	2.665	3.715	6.150	0.433
**Resting tremor**	**Tremor Frequency**	3.163	4.301	3.668	0.862
**Finger tap**	**Log10(Tap Interval Change)**	1.423	2.459	-1.491	1.649
**Finger tap**	**Log10(Tap Interval Symmetry)**	0.828	1.21	-1.058	1.144
**Finger tap**	**Log10(Tap Regularity)**	0.425	0.663	-0.916	0.724
**Finger tap**	**Tap Correctness**	0.139	0.23	0.871	0.264
**Finger tap**	**Tap Count**	27.844	46.124	148.364	0.311
**Finger tap**	**Tap Positional Accuracy**	4.722	6.251	10.333	0.605
**Finger tap**	**Tap Speed**	1.514	2.432	6.434	0.378
